# *Salishicetus meadi*, a new aetiocetid from the late Oligocene of Washington State and implications for feeding transitions in early mysticete evolution

**DOI:** 10.1098/rsos.172336

**Published:** 2018-04-18

**Authors:** Carlos Mauricio Peredo, Nicholas D. Pyenson

**Affiliations:** 1 Department of Environmental Science and Policy, George Mason University, Fairfax VA, USA; 2 Department of Paleobiology, National Museum of Natural History, Washington DC, USA; 3 Departments of Mammalogy and Paleontology, Burke Museum of Natural History and Culture, Seattle WA, USA

**Keywords:** Aetiocetidae, filter feeding, suction feeding, Mammalia, Mysticeti

## Abstract

Living baleen whales, or Mysticeti, lack teeth and instead feed using keratinous baleen plates to sieve prey-laden water. This feeding strategy is profoundly different from that of their toothed ancestors, which processed prey using the differentiated dentition characteristic of mammals. The fossil record of mysticetes reveals stem members that include extinct taxa with dentition, illuminating the morphological states that preceded the loss of teeth and the subsequent origin of baleen. The relationships among stem mysticetes, including putative clades such as Mammalodontidae and Aetiocetidae, remain debatable. Aetiocetids are among the more species-rich clade of stem mysticetes, and known only from fossil localities along the North Pacific coastline. Here, we report a new aetiocetid, *Salishicetus meadi* gen. et sp. nov, from the late Oligocene of Washington State, USA. *Salishicetus* preserves a near-complete lower dentition with extensive occlusal wear, indicating that it processed prey using shearing cheek teeth in the same way as its stem cetacean ancestors. Using a matrix with all known species of aetiocetids, we recover a monophyletic Aetiocetidae, crownward of a basal clade of Mammalodontidae. The description of *Salishicetus* resolves phylogenetic relationships among aetiocetids, which provides a basis for reconstructing ancestral feeding morphology along the stem leading to crown Mysticeti.

## 1. Introduction

Today's living cetaceans represent two major clades that possess fundamentally different innovations related to feeding underwater: Mysticeti, or baleen whales, which filter-feed; and Odontoceti, or toothed whales, which echolocate underwater to find their prey [1,2]. Both innovations are unique to these clades, and are probably important components of the diversification of these two groups since their late Eocene origin, subsequent to the initial land--sea transition in stem cetaceans. The origins of both evolutionary innovations (i.e. filter-feeding and echolocation) in crown cetaceans remain unclear. Recently, a bevy of new fossil material from the stem lineage of Mysticeti has provided a new basis for proposing different hypotheses about the evolution of filter-feeding. Embryological and fossil data converge in showing that the earliest baleen whales (i.e. stem mysticetes) possessed teeth (and lacked baleen); the subsequent transformation to edentulous baleen-bearing mysticetes from this ancestral state is among the more enigmatic questions in cetacean evolution (table 1).

**Table 1.** Measurements of *S. meadi* (UWBM 50004) in mm.

	mm
*skull*	
maximum width as preserved	235
width from midline to right lateral margin	180
width from midline to lateral border of exoccipital	120
length of zygomatic process	96
maximum width of foramen magnum	48
maximum height of foramen magnum	38
maximum height of occipital condyle (right)	61
maximum width of occipital condyle (right)	35
width of base of postglenoid process	54
width of glenoid fossa	75
width of external acoustic meatus	12
*periotic*	
length of anterior process	18
length of cochlear process	28
length of posterior process	32
maximum width of periotic	24
*bulla*	
anteroposterior length	60
maximum transverse width	41
anteroposterior length of tympanic cavity	29
transverse width of medial lobe	18
transverse width of lateral lobe	15
*right mandible*	
anteroposterior length as preserved	232
height at distal end	43
height between pc2–pc3	39
transverse width at distal end	15
transverse width between pc2–pc3	23
*left mandible*	
anterposterior length as preserved	361
height between pc3–pc4	38
height at pc7	47
height at anterior termination of coronoid process	67
transverse width at pc3–pc4	22
transverse width at pc7	24
transverse width at anterior termination of coronoid process	24

Aetiocetidae represent a clade of stem mysticetes that possessed teeth and have historically been valuable in understanding these transitional states in baleen whale evolution. Aetiocetids have been found in Oligocene age rocks along the North Pacific Ocean [3,4]. Initially, aetiocetids were interpreted as archaeocete or stem cetaceans [4,5]. However, Van Valen [6] argued for a mysticete identity, which has been confirmed by multiple phylogenetic analyses [3,7,8]. As stem Mysticeti, aetiocetids have been interpreted as potential intermediate forms between their toothed ancestors from the Eocene [9,10] and baleen bearing, edentulous mysticetes. Consequently, questions about their feeding morphology and their role in the evolution of filter feeding have come under scrutiny. Authors have proposed that aetiocetids were raptorial feeding [3,11], filter feeding assisted by proto-baleen [8,12] and suction feeding [13]. Peredo *et al*. [14] provides a full review and framework of these feeding hypotheses across stem Mysticeti.

Interestingly, the phylogenetic relationships among aetiocetids and their position relative to other stem mysticete clades (such as the Mammalodontidae and Eomysticetidae) are inconsistently resolved across different analyses. Some phylogenies [8,15] recover aetiocetids crown-ward of mammalodontids, while others recover a monophyletic Mammalodontidae + Aetiocetidae [7,11,16]. This inconsistency is problematic for interpreting aetiocetid feeding morphology as it relates to hypotheses for the loss of teeth, origin of baleen or the evolution of filter feeding [14]. Given the broad diversity of feeding morphology observed in stem mysticetes, it is critical to reconcile their phylogenetic relationships. Absent a robust phylogenetic hypothesis, the sequence of transitional morphological states connecting crown mysticetes to stem cetaceans remains obscure—i.e. any single inferred feeding mode lacks the phylogenetic context necessary to test explicit hypotheses related to the evolution of mysticete feeding.

Here we describe a new genus and species of aetiocetid with a partial basicranium, a complete tympanoperiotic complex, elements of both dentaries, and a nearly complete lower dentition. We present, to our knowledge, the first phylogenetic analysis to include every species of aetiocetid, including both species of *Fucaia* Marx, Tsai, and Fordyce 2015 [11] and the rarely coded *Chonecetus sookensis* Russell 1968 [5]. In doing so, we revise aetiocetid taxonomy and comment on inter-relationships within Aetiocetidae. Finally, we discuss the breadth of feeding morphologies across aetiocetids within this new phylogenetic context and comment on their implications for the evolution of filter feeding in Mysticeti.

## 2. Material and methods

### 2.1. Digital methods

We scanned the holotype skull and mandibles of *Salishicetus meadi* using Nikon Metrology's combined 225/450 kV microfocus X-ray and computed tomography (CT) walk-in vault system at National Technical Systems in Belcamp, Maryland, USA, with a slice thickness of 0.03 mm. The holotype skull was mounted vertically in the scanner with the posterior side down to minimize scanning width.

The holotype bulla and isolated teeth were scanned using Nikon Metrology's 225 kV microfocus X-ray CT cabinet system, also at National Technical Systems, with a slice thickness of 0.03 mm. DICOM files from these scans were processed in Mimics (Materialise NV, Leuven, Belgium) to create a three-dimensional model of the UWBM 50004 cranium, mandibles, bulla and isolated teeth. The three-dimensional models are archived at Zenodo (http://zenodo.org) at the following (doi:10.5281/zenodo.834321).

### 2.2. Phylogenetic analysis

To test the phylogenetic position of *S. meadi*, we coded UWBM 50004 into the Peredo & Uhen [17] matrix, which was modified from Boessenecker & Fordyce [18]. We added the character state coding for *Matapanui waihao* Boessenecker and Fordyce 2016 [19] and, in an effort to test the monophyly of Aetiocetidae, coded *Fucaia buelli*, *Morawanocetus yabukii* Kimura and Barnes [3], *C. sookensis*, *Aetiocetus tomitai* Kimura and Barnes [3], and three unnamed Oligocene mysticetes: USNM 314627, UWBM 82941 and UWBM 87135, in addition to *S. meadi*. The final matrix, which includes 86 operational taxonomic units and 363 total characters, is the most robust matrix for mysticetes by both measures. We performed a cladistic analysis in TNT* [20] using unordered and equally weighted characters.

Analysis was conducted using the ‘traditional search' option and included 10 000 random addition sequences, saving 10 trees per replicate. The analysis resulted in 614 most parsimonious trees with a best score of 1587 steps. The final matrix is available in the electronic supplementary material. A 50% majority rule consensus tree is figured here, and support for relevant clades is discussed in §5.2.

### 2.3. Specimens observed

*Aetiocetus cotylalveus* (USNM25210), *Aetiocetus polydentatus* (cast of AMP 12), *Aetiocetus tomitai* (cast of AMP 2), *Aetiocetus weltoni* (UCMP 122900), *Chonecetus sookensis* (NMC VP12095), *Fucaia buelli* (UWBM 84024), *Fucaia goedertorum* (LACM 131146), *Janjucetus hunderi* (NMV P216929), *Mammalodon colliveri* (NMV P199986), *Morawanocetus yabukii* (cast of AMP 1), *Sitsqwayk cornishorum* (UWBM 82916), UWBM 82941, UWBM 87135.

### 2.4. Institutional abbreviations

AMP, Ashoro Museum of Paleontology; LACM, Natural History Museum of Los Angeles County; NMC, National Museum of Canada; NMV, National Museum Victoria; UCMP, University of California Museum of Paleontology; UWBM, Burke Museum of Natural History and Culture; USNM, Smithsonian National Museum of Natural History.

## 3. Systematic palaeontology

Cetacea Brisson, 1762

Pelagiceti Uhen, 2007

Neoceti Fordyce & de Muizon, 2001

Mysticeti Gray, 1864

Aetiocetidae Emlong, 1966

*Salishicetus*, gen. nov.

**LSID.** urn:lsid:zoobank.org:pub:46C7BBBB-480F-48B8-9094-96AF5B73D6EB

**Type species:** *Salishicetus meadi,* gen. et sp. nov.

**Etymology.** Salish-, which reflects the type specimen's provenance near the Salish Sea, a geographical area that includes Puget Sound and honours the tribes of Washington State and First Nations of British Columbia that are historically connected to the region, otherwise known as the Coast Salish or Salish Straits people; and ketos (Gr.) for cetaceans. The genus epithet also draws on inspiration from *Salishia* Clarkston and Saunders 2012 [21], a coastal red seaweed from British Columbia and another stem mysticete from the Olympic Peninsula of Washington State, *Fucaia* [11], named after the Juan de Fuca Strait.

**Diagnosis.** Same as that of the species.

*Salishicetus meadi*, sp. nov. (figures 1–9; table 1)

**Figure 1.**
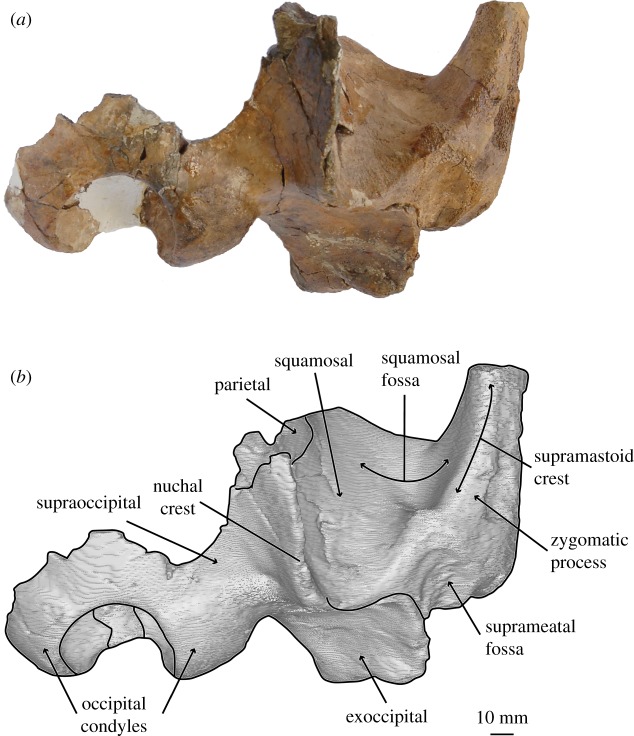
(*a*) Holotype skull of *S. meadi* (UWBM 50004) in dorsal view; (*b*) line art superimposed on 3D model of holotype skull. Dashed symbols represent plaster reconstruction on the specimen.

**Figure 2.**
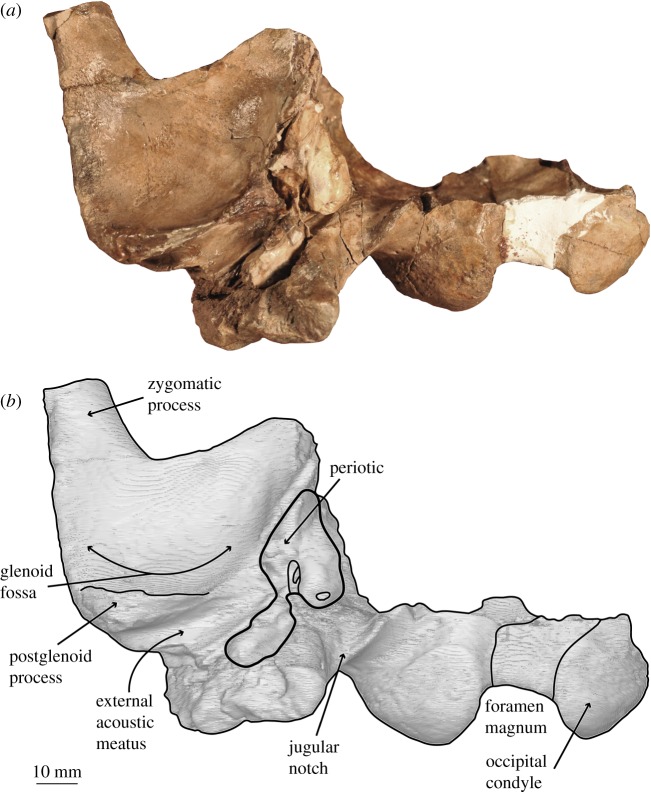
(*a*) Holotype skull of *S. meadi* (UWBM 50004) in ventral view; (*b*) line art superimposed on 3D model of holotype skull. Dashed symbols represent plaster reconstruction on the specimen.

**Figure 3.**
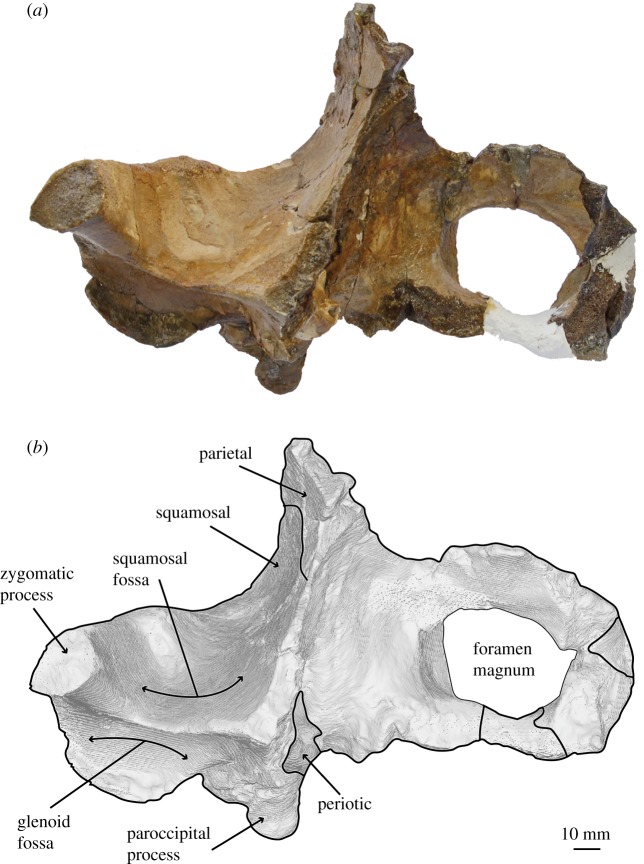
(*a*) Holotype skull of *S. meadi* (UWBM 50004) in anterior view; (*b*) line art superimposed on 3D model of holotype skull. Dashed symbols represent plaster reconstruction on the specimen.

**Figure 4.**
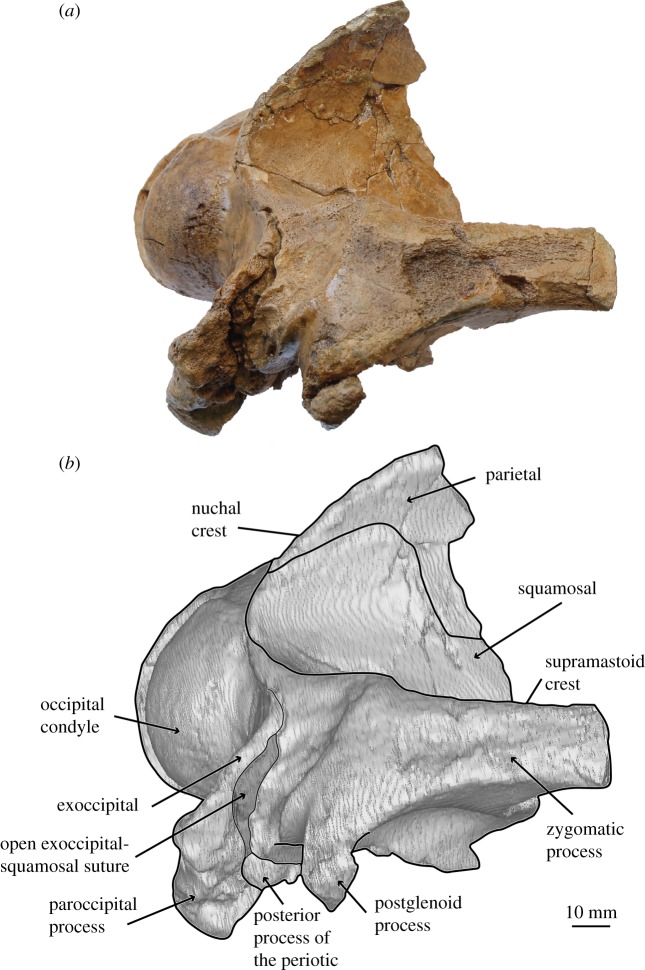
(*a*) Holotype skull of *S. meadi* (UWBM 50004) in lateral view; (*b*) line art superimposed on 3D model of holotype skull.

**Figure 5.**
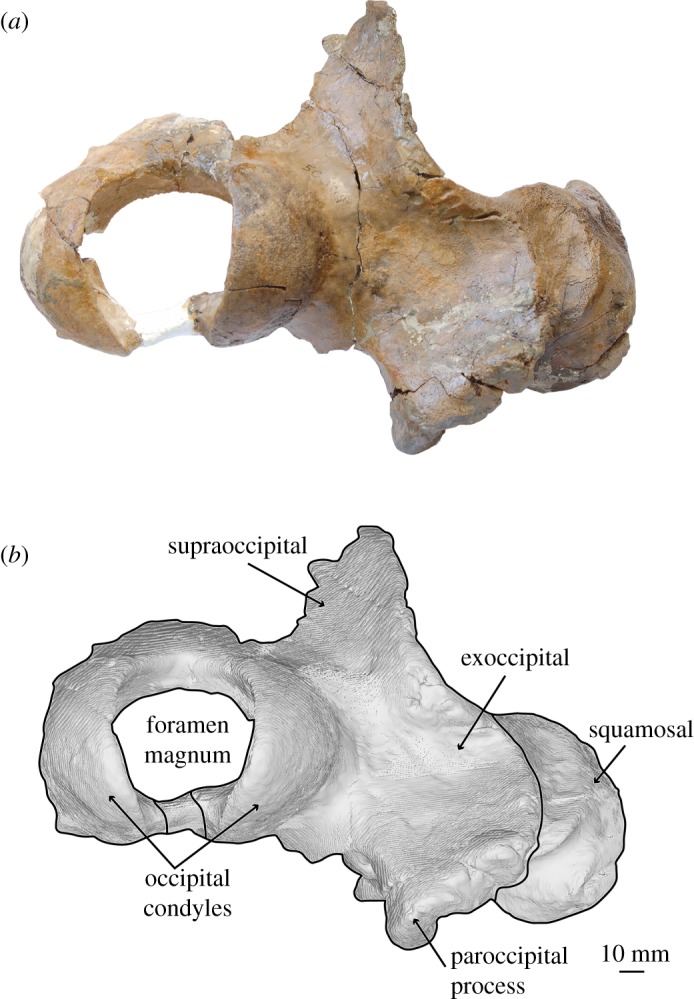
(*a*) Holotype skull of *S. meadi* (UWBM 50004) in posterior view; (*b*) line art superimposed on 3D model of holotype skull. Dashed symbols represent plaster reconstruction on the specimen.

**Figure 6.**
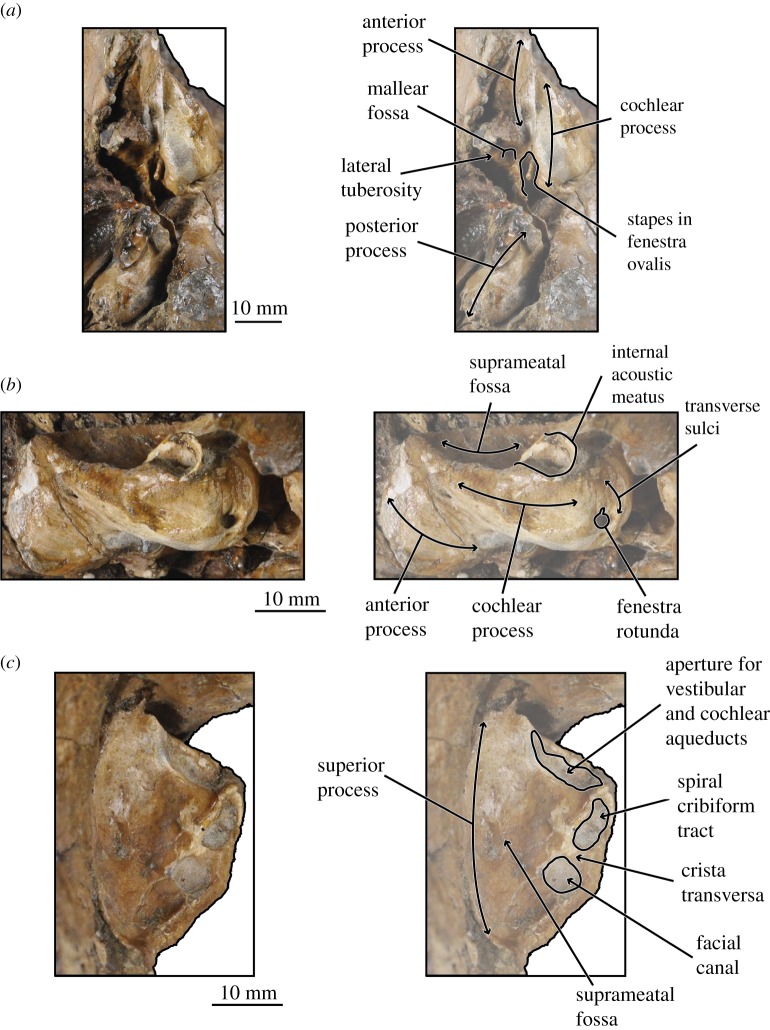
Holotype periotic of *S. meadi* (UWBM 50004) in (*a*) ventral view; (*b*) medial view; and (*c*) dorsal view.

**Figure 7.**
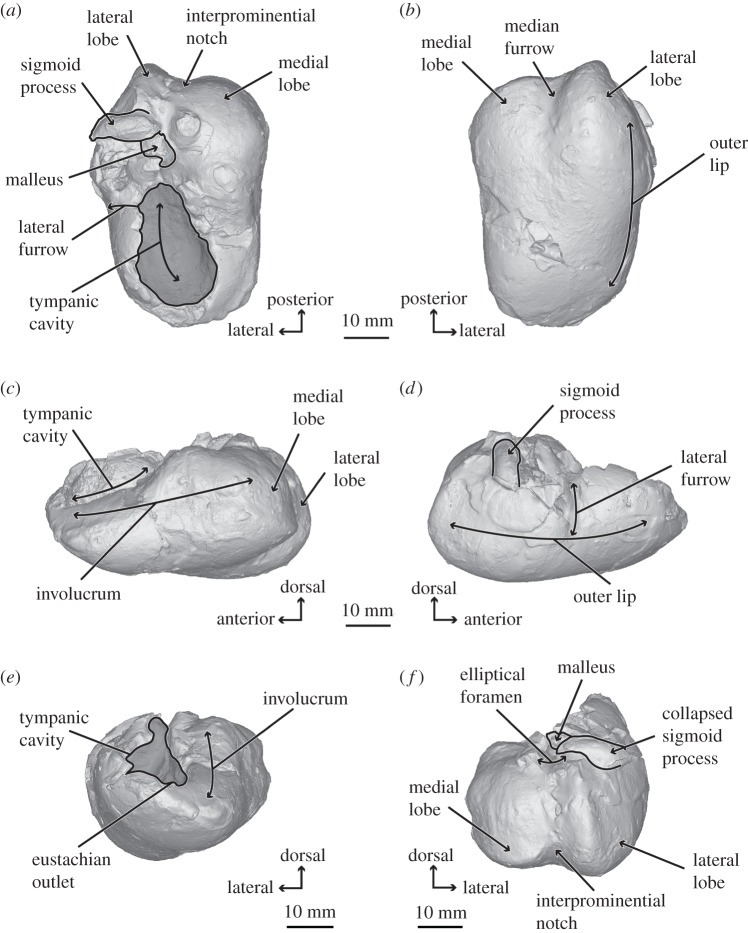
Three-dimensional model of holotype bulla of *S. meadi* (UWBM 50004) in (*a*) dorsal; (*b*) ventral; (*c*) medial; (*d*) lateral; (*e*) anterior; and (*f*) posterior view.

**Figure 8.**
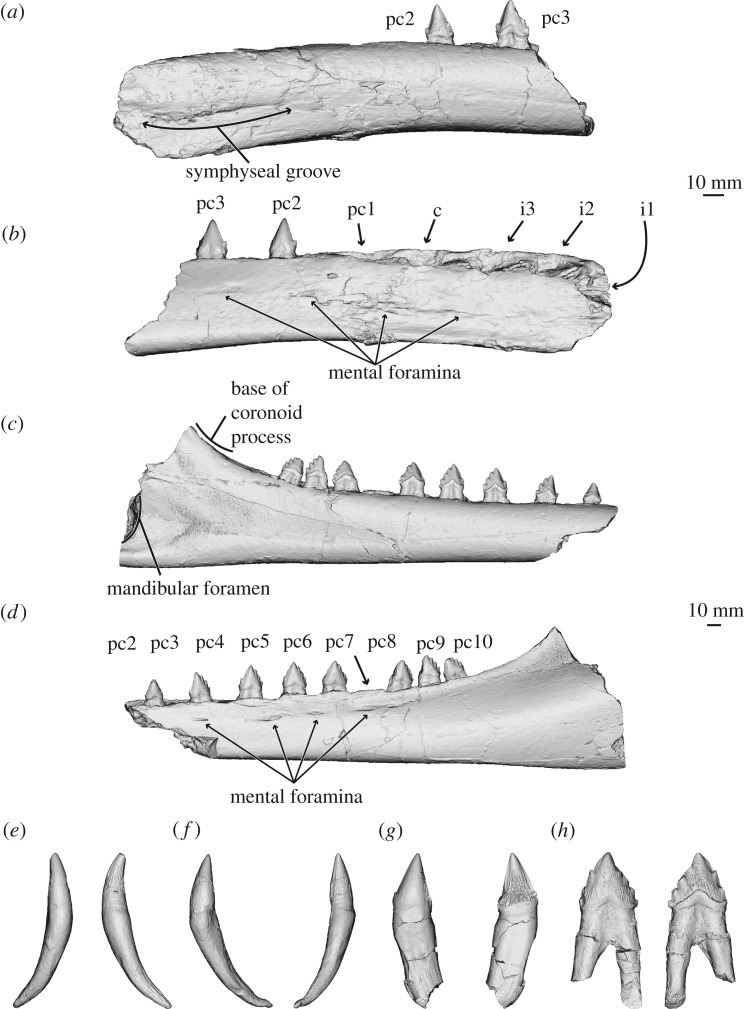
Three-dimensional model of holotype mandibles and isolated teeth of *S. meadi* (UWBM 50004). Right mandible in (*a*) medial and (*b*) lateral view. Left mandible in (*c*) medial and (*d*) lateral view. (*e*) Possible right canine in labial and lingual view; (*f*) possible left i3 in labial and lingual view; (*g*) possible left pc1 in labial and lingual view; (*h*) left pc7 in labial and lingual view.

**Figure 9.**
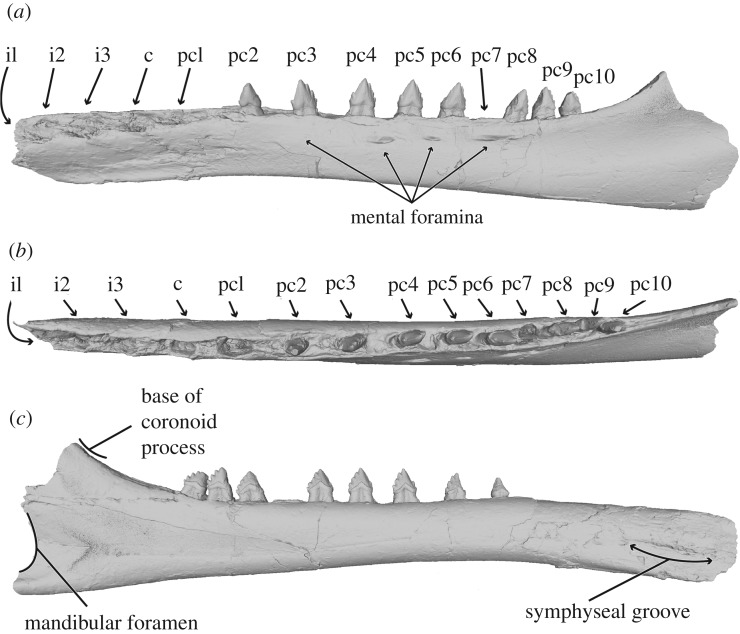
Composite three-dimensional reconstruction of the left mandible of *Salishicetus meadi* using a mirror of the right mandible in (*a*) lateral; (*b*) dorsal; and (*c*) medial view.

**LSID.** urn:lsid:zoobank.org:pub:46C7BBBB-480F-48B8-9094-96AF5B73D6EB

**Etymology.** The specific epithet honours Dr James G. Mead, native son of Washington State, for his lifetime of contribution to the study of cetacean morphology, ecology, historiography, his stewardship of natural history collections, his support of the Smithsonian Institution, and mentoring many generations of students and researchers.

**Holotype.** UWBM 50004, consisting of a basicranium, portions of the right and left mandible with a near-complete dentition, and a right tympanoperiotic.

**Type Locality.** UWBM Locality number C0002; Helsing Junction, Thurston County, Washington State, USA. UWBM 50004 discovered and collected at 46°48′21.69 N, 123°, 07′08.23 W along the south bank of the Chehalis River between the river bank and Lundeen Road SW. Type locality is approximately 4 km southwest of the town of Rochester, Washington State. Associated fauna recovered at the type locality includes *Heptranchias* (UWBM 50014 and UWBM 60692). This locality is Palaeobiology Database locality number 185376.

**Formation.** Lincoln Creek Formation.

**Age.** What is now the Lincoln Creek Formation was originally identified in Thurston and Lincoln counties by Weaver [22] as the Lincoln Formation and regarded the entire outcrop as Oligocene in age. This name was preoccupied by Eocene rocks in Colorado, prompting Beikman *et al*. [23] to use the name Lincoln Creek Formation instead. These latter authors note that, although the name Lincoln Creek Formation is taken from Lincoln Creek in Lewis County, Washington, rocks in this area are minimally exposed. Instead, these authors designated the type section of the Lincoln Creek Formation as the rocks exposed along the Chehalis River from Galvin to Helsing Junction. These authors considered the Lincoln Creek Formation to range from late Eocene to early Miocene. Specifically, they identify the type section at Helsing Junction as late Oligocene in age and correlated with the type Blakeley Formation of Weaver [22]. This late Oligocene age is consistent with biostratigraphic ages from nearby fossil mollusc localities reported by Miller [24] and Schenck [25].

**Diagnosis.** *Salishicetus* is an aetiocetid identified as a unique taxon in this analysis by the following combination of autapomorphies: transverse sulci and ridges along the dorsal margin of the fenestra rotunda of the periotic; an apex of the mandibular terminus positioned halfway between the dorsal and ventral margins; and a spear-shaped mandibular terminus in lateral view. A full differential diagnosis of the genus is listed in the Discussion, following a taxonomic revision to problematic genera in Aetiocetidae.

## 4. Description

### 4.1. Overview

Anatomical terminology follows Mead & Fordyce [26]. Description of the skull refers to either the right or left side, whichever is most complete, unless otherwise stated. Any asymmetry observed is noted. The mandibles of *S. meadi* are described separately herein, although together they represent nearly a complete mandible.

### 4.2. Cranium

The holotype skull of *S. meadi* (UWBM 50004) consists of an incomplete basicranium (figures 1–5) with the periotic (figure 6) preserved in articulation and the tympanic bulla preserved in isolation (figure 7). The posteriormost portions of the supraoccipital shield are preserved along with the right nuchal crest, the right squamosal and zygomatic process and the right exoccipital. Both occipital condyles are preserved, though no basioccipital is present. The temporal wall preserves the dorsal and posteriormost margin of the parietal.

### 4.3. Squamosal

The right squamosal is preserved in the temporal wall and extending down to the squamosal fossa, and includes the right zygomatic process. In the temporal wall, the anteriormost surface of the squamosal is concave so that in dorsal view it is hidden by the overhanging nuchal crest. The squamosal-parietal suture preserves as a tight articulation between the two bones but is not fused, resembling the condition seen in *Aetiocetus cotylalveus* Emlong 1966 [4]. This suture begins posteriorly at the nuchal crest and extends horizontally to a position in line with the midpoint of the zygomatic process. At this point, the suture turns nearly ninety degrees ventrally and extends to the floor of the squamosal fossa. This morphology differs from the condition seen in *A. cotylalveus*, which has a sinusoidal squamosal-parietal suture that extends dorsal for some length before turning ventrally. *Aetiocetus cotylalveus* has a deep, lobate projection of the squamosal pinching the parietal near the nuchal crest. *Salishicetus meadi* instead has a fat dorsal margin of the squamosal that angles sharply at its anterior termination.

As the squamosal transitions laterally from the temporal wall towards the squamosal fossa it becomes less steep, though it never becomes horizontal as in *A. cotylalveus*. As a result, the squamosal fossa in *Salishicetus* bears a heavy anterior and somewhat lateral inclination, in stark contrast to the dorsally facing squamosal fossa of *A. cotylalveus*. Consequently, the squamosal fossa in *Salishicetus* recedes posteriorly and opens facing anteriorly instead of dorsally as in many stem mysticetes.

On the dorsal surface, the nuchal crest transitions smoothly into the supramastoid crest as it passes from the braincase to the lateral region of the squamosal. It remains distinct, if somewhat blunt and rounded, as it moves anteriorly over the body of the squamosal but becomes almost indistinct as it transitions onto the zygomatic process. Consequently, the dorsal surface of the zygomatic in *Salishicetus* is gently rounded in cross-section.

Posteriorly, the squamosal contacts the exoccipital at a loose suture. Though the bones are in tight articulation deep within the skull, at the surface the two bones do not fully articulate. This gap appears as somewhat of an open suture that is a deep trough for most of its length. Immediately anterior to this suture lies the main body of the squamosal dorsal to the postglenoid process and the glenoid fossa. At this level, the squamosal is dorsoventrally thickened and robust. The dorsal margin is extremely elongate compared to that of *A. cotylalveus* so that there is an overall robust and pronounced body of the squamosal, at the base of the zygomatic process, that is separate and distinct from the squamosal basin.

In ventral view, the squamosal preserves a dorsoventrally deep and anteroposteriorly broad external acoustic meatus that originates at the lateral margin of the periotic and moves posterolaterally to the lateral margin of the skull. The margins of the squamosal adjacent to the anterior process of the periotic are incomplete and do not inform about the morphology of the falciform process. The external acoustic meatus is bound posteriorly by the paraoccipital process of the exoccipital and anteriorly by the postglenoid process of the squamosal. The postglenoid process is angled ventrally with minimal anterior hooking or recurvature. At its base, the postglenoid process is broad both transversely and anteroposteriorly but it becomes anteroposteriorly thin moving ventrally so that at its apex it is a thin, blade-like structure. Overall, the postglenoid process is dorsoventrally short compared to other aetiocetids, resulting in a comparatively shallow glenoid fossa.

The zygomatic process is preserved for some distance, though its distal tip is broken and missing. In lateral view, it preserves a straight dorsal margin and a gently concave ventral margin, giving it a gentle dorsal arch. In dorsal view the zygomatic process bears a slight lateral deflection. Though the distal tip is broken, there is no evidence to suggest any globular or bulbous inflation as seen in the zygomatic process of *A. cotylalveus*.

### 4.4. Supraoccipital

The right lateral margin of the supraoccipital is preserved to about the level of the midpoint of the zygomatic process. At this level, the supraoccipital slopes steeply up the nuchal crest, which preserves a sharp keel. The nuchal crest is deflected laterally so that it substantially overhangs the temporal wall. This condition differs from the nuchal crest of *A. cotylalveus* which preserves only a slight lateral deflection of the nuchal crest; *Chonecetus* spp. show little to no such overhang. Posteriorly, the supraoccipital descends steeply towards the occipital condyles where it contacts the exoccipital. The suture with the exoccipital is fully fused. Though badly broken and poorly preserved, the supraoccipital is overall steeply inclined dorsally moving anteriorly. This condition is drastically different from *A. cotylalveus* and *Aetiocetus weltoni* Barnes and Kimura [3], which preserve a shallower anterior inclination of the supraoccipital, but appears more similar to *Chonecetus* spp. and *Fucaia goedertorum* Barnes and Furusawa [3].

### 4.5. Exoccipital

The exoccipital is a dorsoventrally tall and transversely wide plate of bone lying lateral to the occipital condyle. Dorsally it contacts the supraoccipital at a suture that is fused and obscured. Laterally and anteriorly it contacts the squamosal at a suture that, in posterior view, is laterally convex so that it creates a rounded lateral margin of the exoccipital. As mentioned above, this suture is tight deep in the skull but loose at the surface so that, superficially, some space exists between the exoccipital and the squamosal. At its lateral margin the exoccipital thickens anteroposteriorly so that it lies well posterior to the posterior termination of the squamosal. In lateral view, the exoccipital is posteriorly concave, so that it preserves as a deep pit or valley between the lateral margin and the occipital condyle.

In posterior view, the shape of medial margin of occipital condyles makes this feature appear semicircular, not reniform, in *Salishicetus*. The medial margins of the occipital condyles are aligned nearly dorsoventrally, with a slight lateral inclination, and are straight, not concave. The foramen magnum is oval in shape, with a curved dorsal margin and the aforementioned straight, laterally deflected lateral margins. The ventral margin of the foramen magnum is not preserved.

At its ventral margin, the exoccipital transitions into the paroccipital process. In lateral view the paroccipital process reaches nearly the same level posteriorly as the occipital condyles. In posterior view it preserves as a triangular ventral projection of the exoccipital that reaches a level far ventral to that of the postglenoid process of the squamosal. The apex is notably deflected medially, so that it has a steep, ventrally oriented medial margin and a gently sloping medioventrally oriented lateral margin. The entire paroccipital process is anteroposteriorly expanded compared to the flat, plate-like exoccipital dorsal to it. Ventrally, the paroccipital process preserves a shallow posterior sinus and, medial to it, a deep jugular notch. This jugular notch is bound laterally by the posterior sinus and medially by a bulbous expansion of the paroccipital process as it reaches its ventral apex.

### 4.6. Periotic

UWBM 50004 preserves a complete right periotic *in situ* (figure 6). The periotic is visible in ventral and medial views, and part of the superior process is visible in dorsal view through the braincase. In ventral view the anterior process is transversely thin and blade-like, and is oriented with a slight medial deflection. It is tightly appressed against the squamosal laterally. The cochlear portion of the periotic is anteroposteriorly elongate and inflated medially. The anterior margin of the cochlear process slopes gently anteriorly so that the transition between the cochlear process and the anterior process is smooth and gradual. By contrast, the posterior margin of the cochlear process is posteriorly convex so that the transition between the cochlear process and the posterior process is sharp and abrupt. These margins serve to give the cochlear process an overall teardrop or pear shape, being broad and inflated posteriorly with a gradually tapering anterior end. The posterior process of the periotic is deflected posterolaterally off the sagittal plane. It terminates at a blunt, rounded posterior apex.

The fenestra ovalis is dorsoventrally deep and is positioned in the posterior half of the cochlear process. It preserves a well-defined, rounded anterior margin but a poorly defined, open posterior margin. Preserved within the fenestra ovalis is the rod-shaped stapes. Anterolateral to the fenestra ovalis is the mallear fossa, which preserves as a shallow pit medial to the lateral tuberosity. The lateral tuberosity can be identified lateral to the mallear fossa and is well developed and expanded into an elongate, pointed tip.

In medial view the anterior process is oriented anteroposteriorly. It preserves a squared off anterior tip with only a very faint dorsal apex. The dorsal margin is relatively flat as it transitions from the anterior process onto the superior process. The ventral margin bears a slight ventral convexity, being gently rounded ventrally at the anterior process and recessed dorsally as it transitions to the cochlear portion. The posterior process is angled posteroventrally at a 45° angle, creating a sharp demarcation between the cochlear and posterior processes. In medial view, much of the posterior process is eclipsed by the paroccipital process of the exoccipital.

The fenestra rotunda is situated on the posterior surface of the cochlear process and orients posteromedially. It is oval in shape, being slightly longer anteroposteriorly than dorsoventrally, and preserves several distinct ridges running from its dorsal margin. The aperture for the vestibular aqueduct and the aperture for the cochlear aqueduct are visible on the dorsal surface of the cochlear process and are confluent, appearing as a single, transversely elongated foramen. Together, the apertures are deep and run nearly the entire transverse width of cochlear process in dorsal view.

In dorsal view, only a small portion of the anterior process is visible. Instead, most of the dorsal exposure of the periotic is the suprameatal fossa, which preserves as a deep basin bound laterally by the lateral margin of the superior process and medially by the internal acoustic meatus. The internal acoustic meatus preserves as two distinct openings: a roughly circular-shaped endocranial opening for the facial canal and an elongated, oval shaped spiral cribiform tract. These two openings are separated by a high crista transversa that is angled posteriorly so that the foramen singulare is obscured in dorsal view.

### 4.7. Tympanic bulla

UWBM 50004 preserves a nearly complete right tympanic bulla (figure 7), though the dorsal margin shows some distortion and diagenetic alteration. In dorsal view the lateral margin of the outer lip is sigmoidal, being convex posteriorly, deeply concave at its midpoint and convex again anteriorly. The sigmoid process is situated near the posterior margin, completely posterior to the tympanic cavity, and is crushed so that it lies on top of and obscuring the conical process. The sigmoid process preserves as a tubular, transversely oriented cylinder. Damage in this region obscures the mallear ridge. The outer posterior pedicle lies posterior to the flattened sigmoid process and appears low and blunt. The broken malleus lies crushed medial and anterior to the sigmoid process. Posteriorly, the inner and outer posterior pedicles are well developed and separated by a deep elliptical foramen. Medially, the involucral ridge demarcates the medial margin of the bulla and continues to the anterior margin. The dorsal surface of the involucrum is smooth and sloping ventromedially away from the tympanic cavity. Anteriorly, the medial margin of the bulla turns in at a 45° angle to create a bluntly rounded anterior margin of the tympanic bulla.

In medial view the overall ventral margin of the bulla is gently convex, slopping dorsally towards its anterior end. The dorsal margin is stepped, being convex and pronounced to a level just anterior to the sigmoid process and then slopes sharply towards the anterior end. The lateral lobe extends further posteriorly than the medial lobe so that the well-developed median furrow is visible in medial view. The lateral lobe bears a globular, convex posterior margin while the medial lobe preserves a flat, dorsoventrally oriented posterior margin. In lateral view, the posterior margin of the lateral lobe is visible as a sharp ridge extending down from the conical process. Anterior to the broken mallear ridge begins a lateral furrow that runs ventrally across the lateral surface, but is shallow compared to the deeper lateral furrow of *F. buelli*. This lateral furrow divides the lateral surface of the bulla into two distinct regions: a posterior region that is ovoid in shape, and an anterior region that is more triangular in shape as it tapers to a point at the anterior margin of the bulla.

In ventral view the bulla preserves a relatively straight, anteroposteriorly oriented medial margin and a gently curved, convex lateral margin. The posterior portion of the ventral surface bears a deep interprominential notch separating the medial and lateral lobes. The interprominential notch is deep, but short, and does not approach the level of the lateral furrow.

In posterior view the outer posterior prominence is transversely thin compared to the inner posterior prominence. The interpromontorial notch is deep and oriented dorsoventrally. At the ventral margin, the medial lobe bears a transverse crest running from the interpromontorial notch around to the medial surface of the bulla. The inner posterior pedicle is faintly visible at the dorsal margin of the inner posterior prominence. The outer posterior pedicle is visible just posterior to the sigmoid process, which has been compressed down over the conical process, elliptical foramen and other underlying features.

### 4.8. Auditory ossicles

The right malleus is *in situ* and is compacted between the collapsed mallear ridge and sigmoid process of the tympanic bulla. The anterior process of the malleus is broken and missing, but the main body is present. It is robust, anteroposteriorly elongated, and tightly appressed to the sigmoid process at its posterior margin.

The right incus is preserved in isolation from the other ear bones. It is somewhat damaged where it articulated with the malleus, obscuring the morphology around the incudomallear joint. However, the body of the incus is well preserved. The crus breve is notably short and pointed at its apex, but appears to be complete based on its tight articulation with the fossa incudis in the periotic. The crus longum is indistinct from the body of the incus, and the lenticular process cannot be distinguished.

The right stapes is preserved *in situ* within the fenestra ovalis. It is narrow in diameter and is angled posteriorly as it exits the fenestra ovalis. The distal head of the stapes is slightly enlarged so that it is broader in diameter than the body of the stapes.

### 4.9. Mandible and dentition

UWBM 50004 presents an unusual opportunity to understand the morphology of the mandible and lower dentition of aetiocetids. It preserves elements of both the left and right mandibles (figure 8), allowing for a reconstruction of the complete postcanine morphology. Each mandible is described separately herein.

The right mandible of UWBM 50004 is represented solely by an anterior fragment, preserving its distal tip. This fragment preserves alveoli for seven teeth, with the two posteriormost teeth preserved *in situ*. Because the cheek teeth of cetaceans are difficult to differentiate, we conservatively label these first seven teeth as three incisors, one canine, and three postcanine teeth.

In lateral view, the right mandible, which is incomplete, preserves a spear-shaped anterior margin terminating in a point at about the centre of the height of the mandible. This morphology differs from *A. weltoni*, which has an anterior termination nearly at the dorsal margin of the mandible. Superficially, only two mental foramina are visible on the right fragment; however, internal morphology revealed via CT scans indicate that five mental foramina are preserved in this part of the mandible; including the left fragment, the entire mandible may possess as many as nine mental foramina total. In medial view, the distal tip preserves a symphyseal groove extending 6 cm from the distal tip to a level just anterior to the canine.

At the distal margin, between the first incisor and the first postcanine, the ventral surface is transversely thinner than the dorsal surface, which widens to accommodate the alveoli. The result is a transversely thin cross section that narrows ventrally. Posterior to the first postcanine tooth, the ventral margin expands transversely and becomes more rounded, creating a more ovate cross section.

The alveolus for the first incisor is positioned at the extreme distal tip and opens with a strong anterior inclination, suggesting a procumbent first incisor. Each subsequent alveolus is increasingly oriented dorsally so that, overall, the three incisors exhibit notable anterior inclination, the canine only very slight anterior inclination, and the postcanine teeth no anterior inclination at all. All first five alveoli are extremely deep and penetrate down to the level of the mandibular canal. The alveoli of the right fragment indicate that the three incisors and the canine are all single rooted. Only a single alveolus for the first postcanine tooth is visible, but it is anteroposteriorly expanded, suggesting two fused roots in a single alveolus lacking an isthmus. The second and third postcanine teeth, which are preserved *in situ*, expose enough of the root above the dorsal margin of the mandible to confirm the teeth as double rooted. The dorsal margin of the right dentary preserves embrasure pits between the first and second, and between the second and third postcanine teeth.

The second postcanine tooth is triangular in shape, with a crown measuring 10 mm across at its base (anteroposteriorly) and 11 mm from the base to the apex. In addition to the central cusp, it bears a single accessory cusp on both the anterior and the posterior sides. In anterior view, all three cusps bear a slight lingual curvature. The lingual surface of the tooth preserves a notable shelf of enamel above the crown line. The labial surface of the tooth preserves no such shelf. Both the labial and lingual surfaces preserve dorsoventral striations running from the base of the crown to the apex.

The third postcanine tooth is similar in shape but is larger overall, with a crown measuring 14 mm wide at the base and 13 mm tall. As the preceding tooth, the third postcanine tooth preserves a single anterior accessory cusp. However, unlike the preceding tooth, it preserves three posterior accessory cusps. These three accessory cusps become increasingly large moving towards the main, central cusp. As with the preceding tooth, all the cusps bear a slight lingual inclination. Similar to the preceding tooth, a shelf of enamel above the crown line preserves only on the lingual surface, and both the labial and lingual surfaces preserve dorsoventral striations. Both of these teeth preserve wear on the posterior margin, with the posterior accessory cusps being notably worn.

The left mandible is especially informative because all of its alveoli preserve teeth. The anteriormost portion of the left dentary begins at the level of the second postcanine tooth. Here, a break in the mandible exposes the roots of the second postcanine tooth, confirming that it is double rooted. The left mandible is complete posterior to this point until its termination just anterior to the coronoid process and the mandibular foramen. The coronoid process, angular process, and articular condyle are all missing. The seventh postcanine tooth was removed during preparation and moulding, but it easily articulates into its original position in the alveolus. The left mandible fragment preserves the nine most proximal teeth, which represent the second through to the tenth postcanine teeth.

In lateral view, the left mandible preserves four mental foramina at the following levels: at the third postcanine tooth, anterior and posterior to the fifth postcanine tooth and at the seventh postcanine tooth. CT data corroborate the external interpretation of four mental foramina. The mental foramina terminate in sulci ranging in length from 12–37 mm, and in thickness by 1–2 mm. In medial view the dorsal and ventral margins are parallel until the level of the fifth postcanine tooth. At this level, the dorsal margin deflects dorsally so that the fifth through to the tenth postcanine teeth are increasingly dorsally elevated. This dorsal deflection continues posterior to the final tooth and rises to the broken coronoid process. At its proximal termination, the left mandible preserves just to the anterior margin of the mandibular foramen, suggesting that the mandibular foramen was broadly rounded at its anterior termination and dorsoventrally expanded. This expanded mandibular foramen is typical of stem cetaceans, odontocetes and stem mysticetes, but differs from crown mysticetes [27,28].

At its distal end, the left mandible is ovate in cross section as the right mandible at the same level. Moving proximally, the dorsal margin narrows, especially posterior to the tenth and final postcanine tooth, resulting in a transversely narrowed cross section at the proximal end, which is reflected in the morphology at the level of the broken coronoid process.

All teeth preserved in the left mandible (postcanine teeth 2–10) are double rooted and oriented dorsally, but bear a similar lingual deflection to those of the right fragment. Moving proximally, there is increasingly less space between teeth, so that the distance between the second and third postcanine teeth measures 16 mm but the distance between the ninth and tenth postcanine teeth measures only 2 mm. Accordingly, embrasure pits are preserved between the second and third, third and fourth, fourth and fifth and perhaps fifth and sixth postcanine teeth. Posterior to this level, the teeth are spaced too close together to accommodate the upper dentition, and no embrasure pits are present.

As with the right dentary, the postcanine teeth preserved in the left mandible are triangular in shape overall. The second postcanine tooth (which is the first tooth preserved in the series) preserves a single, small accessory cusp on both the anterior and posterior sides of the main, central cusp. The third and fourth postcanine teeth each preserve a single anterior accessory cusp and three posterior accessory cusps. The fifth postcanine tooth preserves a single anterior accessory cusp and four posterior accessory cusps. The sixth and seventh postcanine teeth each preserve two anterior accessory cusps and four posterior accessory cusps. The eighth through to the tenth postcanine teeth are each damaged on their anterior margins but are consistent in overall shape and appearance with the two preceding teeth. Each of these latter teeth preserves four posterior accessory cusps and we consider it likely that they also preserved two anterior accessory cusps prior to wear, in life. The teeth in the left mandible increase in size posteriorly, peaking with the seventh postcanine tooth, and then diminishing posterior in the sequence. All of the teeth preserve the lingual shelf of enamel noted in the teeth of the right dentary, and no such structure exists on the labial surface. Similar to the right fragment, all of the teeth bear the dorsoventral striations on both the lingual and labial faces of the enamel. Postcanine teeth two and three preserve little to no wear of the enamel, teeth four through to seven preserve the heaviest wear on the posterior margins, and teeth eight through to 10 preserve the heaviest wear on the anterior margins. In each case, wear is heaviest on the accessory cusps and wear to the central cusp is minimal or nonexistent.

In addition to the teeth preserved in the dentaries, three teeth were recovered in isolation and are here described separately. The first is tentatively identified as the left, lower first postcanine tooth. Overall it is similar in appearance to the second postcanine tooth on the left side, though it is slightly smaller. It also preserves a single accessory cusp on both the anterior and posterior sides, and preserves little or no wear on the enamel. It too preserves a lingual shelf of enamel, though it is much less pronounced than that seen in any of the previously described postcanine teeth. Similarly, it preserves both lingual and labial dorsoventral striations, though they are less elaborate, particularly on the labial side, than in the previously described postcanine teeth. Although it is double rooted, the roots are tightly fused and appear confluent; this is consistent with the right fragment which preserves only a single, expanded alveolus for the first postcanine tooth.

The second isolated tooth is tentatively identified here as the lower left third incisor. This tooth is incisiform in overall shape, being much more conical and less triangular than the previously described cheek teeth. No accessory cusps are present and the single, main cusp is much taller overall compared to its anteroposterior width. As with the other teeth described, this tooth bears a lingual deflection at its apex and dorsoventral striations across the enamel. Unlike the other described teeth, no enamel shelf is present on the lingual surface. Though its position as the third incisor is tentative, its assignment to the left dentary is bolstered by the fact that it does not fit any of the preserved alveoli in the right dentary. This tooth is also unique in that it preserves a complete root, which measures 45 mm in length compared to the 19 mm length of the crown.

The third and final isolated tooth is tentatively identified here as the lower right canine. This tooth is conical and incisiform in shape, preserves little wear, and no lingual enamel shelf. As with the other described teeth, a lingual inflection at the apex is present, though less so than with the other described teeth. Both labial and lingual striations are present, though are much more pronounced on the lingual surface. The tooth preserves a single, complete root, which measures 40 mm in length compared to the 18 mm long crown.

### 4.10. Vertebra

A complete vertebra belonging to UWBM 50004 is preserved and here interpreted as either the first or second thoracic vertebra. The centrum is oval in overall shape, being slightly transversely wider than dorsoventrally tall. In cranial view, the dorsal margin of the centrum is flat and transversely oriented so that the neural canal has a flat ventral margin. The neural arch sits low, with a blunted apex so that the neural canal rises to only a modest apex at its dorsal margin. The neural canal echoes the centrum in being transversely wider than dorsoventrally tall. Overall it is vaguely pear-shaped with its transverse widening, flat ventral margin, and blunt apex at its dorsal margin.

The right transverse process is badly broken, but the left is complete. The left transverse process is flattened dorsoventrally and broad anteroposteriorly, so that its anterior margin terminates in a flat, sharp, blade. At its distal tip, the left transverse process expands into a swollen knob. In cranial view, the transverse processes extend dorsolaterally from the transverse plane at a 45° angle. In cranial view the neural spine begins at the dorsal margin of the neural canal. It bears a sharp, blade-like keel at its anterior face. Its lateral margins extend posterolaterally from this keel so that its total transverse width expands posteriorly. The dorsal apex of the neural spine is not preserved.

In lateral view, the centrum is positioned directly beneath the neural spine, which is similar in anteroposterior length as the centrum. What is preserved of the neural spine is directed dorsally with no evidence for a posterior deflection as seen in the neural spines of *Dorudon*. The transverse processes bear a notable anterior deflection so that their distal terminations are well anterior to the centrum, where they would have articulated with the anterior vertebra. The intervertebral foramen is anteroposteriorly deep and dorsoventrally tall, with a rounded anterior margin so that it is overall C-shaped. The left transverse process preserves a facet for the tuberculum of a rib. The posterior margin of the lateral borders of the centrum each preserve a demifacet for the capitulum of a rib. The lateral margins of the centrum are anteroposteriorly concave, so that the centrum is transversely broadest at its anterior and posterior margins and narrower in the middle, giving it an hourglass shape. The postzyapophysis extends posteriorly from the neural arch and roofs the intervertebral foramen. In lateral view it bears a slight posterodorsal angle.

In caudal view the posterior face of the neural arch and the neural spine bear deep concavities. This results in lateral margins that extend posteriorly beyond medial portions that preserve as deep valleys in the posterior face of the neural arch and neural spine. This morphology is likely to accommodate the sharp, expanded blade-like keep preserved on the anterior margin of the neural spine.

A poorly preserved transverse process and minimal portion of a centrum, as well as several other isolated vertebral elements belonging to UWBM 50004 are also preserved. Unfortunately, these elements are unidentifiable and are largely uninformative with regards to the vertebral morphology of *S. meadi*.

### 4.11. Ontogeny and body size

Skeletal maturity was assessed based on established osteological indicators such as the fusion of cranial sutures and textural surface of the occipital condyles [29]. UWBM preserves clearly distinguishable, closed sutures, with one exception: the exoccipital-squamosal suture, which preserves some separation. However, this suture is also broadly open for some length in *A. cotylalveus*. Thus, it is unclear whether this condition is an ontogenetic trait unique to *S. meadi* or perhaps is broadly observed in aetiocetids. The occipital condyles are entirely smooth and devoid of any pitting typically indicative of an early ontogenetic age. Based on this morphology, we interpret UWBM 50004 as belonging to an osteologically mature individual.

We used the stem mysticete body size equation for bizygomatic width from Pyenson & Sponberg [29] to estimate the total body length of *S. meadi*. We calculated a bizygomatic width by doubling a straight-line orthogonal measurement of skull width from one side, using large calipers to measure a midpoint above the occipital condyle to lateral margin of the zygomatic process of the squamosal. Based on these data using a bizygomatic width of 32.6 cm, *S. meadi* had an approximate total length of 3.09 m. This method probably provides a slight underestimate because the right zygomatic process is not complete and appears to have continued to flare laterally by a few millimeters. Nevertheless, this value is approximately 10% larger than Pyenson & Sponberg [29] estimated for *A. cotylalveus* using bizygomatic width.

## 5. Discussion

### 5.1. Reconstruction of the mandible and dentition

Several overlapping morphological features shared by both the right and left mandibles of UWBM 50004 facilitate a reconstruction of the complete dental row (figure 9). The first and second postcanine teeth on the right fragment match the anteriormost teeth on the left incomplete mandible in both anteroposterior crown lengths and crown heights. In general, the first two postcanine teeth are smaller than the subsequent postcanine teeth. The anteriormost postcanine tooth on the left mandible preserves a single anterior and posterior accessory cusp, as does the second postcanine tooth in the right fragment. Similarly, the subsequent tooth on the left mandible preserves a single anterior accessory cusp and three posterior accessory cusps; the same morphology is observed in the right third postcanine tooth. Similarly, these teeth are consistent in number of roots and overall size across both mandibles.

In addition to these dental similarities, the shape of the mandibles in transverse section through the levels of the second and third postcanine teeth are similar in preserving a flat labial surface and a concave lingual surface, further implying homology of these regions. Thus, the overall morphology across the two mandibles strongly suggests that the left mandible begins with the second postcanine tooth, implying that *S. meadi* preserved 10 postcanine teeth in total.

Three isolated teeth were found in association with UWBM 50004. The first tooth preserves a crown with edged, small accessory cusps (one anterior and one posterior). This tooth is double rooted, though the two roots are tightly appressed and fused. Given these characteristics, this tooth is probably a first postcanine tooth. Its curvature and anatomical side imply either a lower left or upper right first postcanine tooth, though neither can be falsified as no alveoli are present in these quadrants.

The other two isolated teeth preserve long, thin roots that taper and curve dramatically. These teeth preserve crowns that are smooth on the labial side and exhibit longitudinal striations on the lingual side; this morphology is found in other aetiocetids and differs notably from that of mammalodontids. Given this morphology, these teeth probably represent incisors or a potential canine. One of the two is here interpreted as the lower left third incisor given that the curvature of its root matches the alveolus for the third incisor on the right dentary; however, its orientation precludes it from belonging to the right tooth row. The other of the two is interpreted as the lower right canine for similar reasons. Though it cannot be asserted with certainty, the tooth in question occludes comfortably in the alveolus for the canine in the right dentary and the overall crown shape is consistent with the morphological patterns observed in the second and third postcanine teeth.

### 5.2. Monophyly of Aetiocetidae

**Figure 10.**
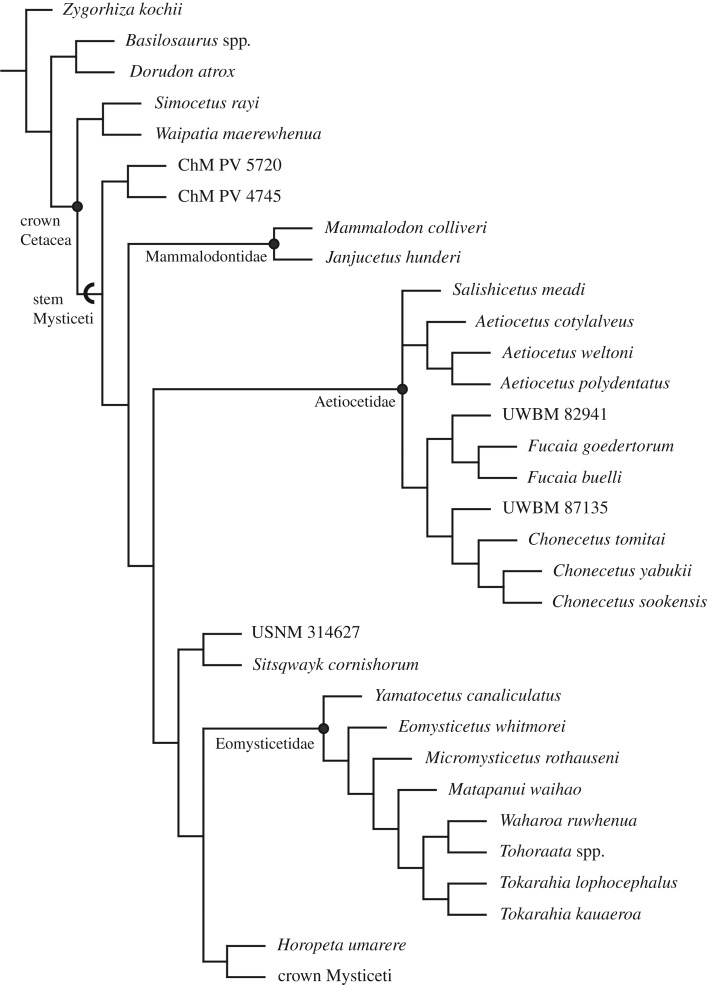
Phylogenetic relationship of *Salishicetus meadi* to other stem Mysticeti. Cladogram shown is a 50% majority rule consensus tree. Crown Mysticeti has been collapsed into a single terminal unit for visualization, but relationships therein are unchanged from Peredo & Uhen [17]. See §2.2 for parameters used in the analysis.

Our analysis (figure 10) differs from previous results using this matrix [17,18,30] in recovering a monophyletic Aetiocetidae, and is consistent with previous results in resolving the Mammalodontidae as a separate clade, basal to Aetiocetidae, unlike results from other matrices [7,11,16]. Here, a monophyletic Aetiocetidae is united by the following synapomorphies: anterior edge of the nasals just anterior to or at the level of the anterior edge of the supraorbital process; lacking a postorbital ridge of the frontal; lacking longitudinal grooves on the posterior bullar facet of the periotic; dorsoventrally deepest part of the anterior process of the periotic lying within the posterior 50% of the anterior process; aperture for the vestibular aqueduct transversely wider than the opening of the facial canal; posteriormost upper tooth anterior to the antorbital notch; and a straight radius lacking anterior curvature.

Despite a monophyletic Aetiocetidae, we do not recover a monophyletic *Aetiocetus* spp., as previously defined. Three of the four species of *Aetiocetus* form a well-supported clade (recovered in 100% of our best score trees), whereas the fourth, *A. tomitai*, consistently groups in a basal branch sister to a clade formed by *C. sookensis* and *M. yabukii* (a result that was also recovered in 100% of best score trees). Accordingly, we consider *A. tomitai* and *M. yabukii* (both from the late Oligocene of Hokkaido, Japan) to belong to *Chonecetus* along with *C. sookensis* (from the late Oligocene of British Columbia, Canada). Here we formally recombine these taxonomic units as *C. tomitai* (nov. comb.) and *C. yabukii* (nov. comb.). It is worth noting, however, that these taxonomic decisions rest on material with limited comparability within Aetiocetidae: *C. sookensis* preserves only 90 of the 363 characters (24.8%) in this matrix—the type and only known specimen consists of an incomplete cranium with a single left periotic *in situ*. Nevertheless, removing *C. sookensis* from the analysis results in nearly the same consensus tree, with the slight difference that presents *C. yabukii* and *C. tomitai* as sister taxa.

Given this taxonomic revision, our analysis returns three distinct subclades within Aetiocetidae: (i) a now monophyletic *Aetiocetus* (100% of best score trees); (ii) a clade of *Fucaia* spp.* *+ *Chonecetus* spp. (85% of best score trees); and (iii) a single lineage representing *S. meadi* (100% of best score trees). The first of these (*Aetiocetus* spp.) is united by the following synapomorphies: premaxillae that are separated along most of the rostrum but medially fused at the anterior tip; an orbitotemporal crest that extends posteriorly onto the parietals; a zygomatic process that is directed anteromedially; a zygomatic process that is expanded anteriorly and posteriorly but constricted in the middle in lateral view; a narrow squamosal lateral to the exoccipital; a dorsally arched squamosal in lateral view; and a horizontal crest on the posterior surface of the medial love of the tympanic bulla. The second is united by the following synapomorphies: premaxillae that face anteriorly at their posterior end; and an external occipital crest that is either blunt or absent. The third clade, which represents only *Salishicetus*, is identified as unique based on the autapomorphies used in the diagnosis (§3). UWBM 82941 resolves as basal to the clade of *Fucaia* spp. and UWBM 87135 resolves as basal to the clade of *Chonecetus* spp. *Salishicetus* is resolved in polytomy with these other sub-clades within Aetiocetidae, which reflects the unique characters that distinguish it from other aetiocetids such as the strongly heterodont cheek teeth with elaborate accessory cusps and the embrasure pits on the mandible.

### 5.3. Feeding morphologies and the evolution of filter feeding

The position of aetiocetids in mysticete phylogeny has led to the interpretation that they represent intermediate forms between macrophagous raptorial predators (i.e. the ancestral condition represented in basilosaurid cetaceans) and bulk filter feeders (as seen in living mysticetes). Understanding the details of this transition from raptorial to filter-feeding along the lineage leading to living mysticetes requires a thorough study of the diversity in morphological states among aetiocetids and other stem mysticetes. For example, some aetiocetids exhibit a simplification of root and cusp count from their ancestral states resulting in dentition similar to that of homodont odontocetes [3]. Other aetiocetids preserve palatal foramina that have been interpreted as proxies for proto-baleen [12]. Most recently Marx *et al*. [13] described an unnamed aetiocetid with dental wear indicative of suction feeding. However, none of the observed morphologies are consistent across all aetiocetids; aetiocetid morphology only bears on the evolution and origin of filter feeding in mysticetes if we can accurately resolve the ancestral condition for the clade. The key question, in this regard, is whether any aspect of aetiocetid feeding morphology bears on the origin of baleen; given the morphology observed in *Salishicetus*, we suggest that such answers lie closer to crown Mysticeti. It is, therefore, worth considering each aspect in more detail.

#### 5.3.1. Dental simplification

Species of *Aetiocetus* exhibit a simplified dentition with single rooted cheek teeth and greatly diminished accessory cusps–so much so that they were originally described as homodont by Barnes *et al*. [3], though we agree with Deméré and Berta [8] that ‘weakly heterodont’ is a more appropriate classification. However, other aetiocetids such as *C. yabukii*, and *F. buelli* preserve double rooted cheek teeth with larger and more elaborate cusps and probably used the dentition for shearing and prey processing [11].

*Salishicetus meadi* is the most strongly heterodont aetiocetid known; the cheek teeth are substantially larger overall than those of *F. buelli*, and bear notably taller crowns. *Chonecetus yabukii* also preserved a single upper cheek tooth that vaguely resembles that of *Fucaia* in morphology [3]. Though no upper dentition is preserved, the extensive wear on the cheek teeth, especially on the accessory cusps, as well as the deep embrasure pits on both dentaries, suggest a tight occlusion for shearing. Because this feeding morphology is observed in more basal mysticetes [10,31], and in three distinct genera of aetiocetids (*Salishicetus*, *Chonecetus* and *Fucaia*), we consider it the most likely ancestral condition. Therefore, we consider the dental simplification of *Aetiocetus* spp. a derived trait unrelated to the loss of dentition in crown Mysticeti.

#### 5.3.2. Suction feeding

Recently, an undescribed aetiocetid from the North Pacific basin (NMV P252567) exhibiting dental wear associated with suction feeding has led to the hypothesis that suction feeding preceded filter feeding [13]. However, the implications of this feeding morphology are difficult to interpret. The earliest known mysticete, *Mystacodon selenensis* Lambert *et al*. [10] also exhibits evidence of suction feeding. Thus, at least three distinct lineages of toothed mysticetes are thought to have been suction feeders (*M. selenensis,* Mammalodontidae and NMV P252567).

We do not disagree with any of the above authors in their interpretation of suction feeding for the respective taxa. However, it is well established that both raptorial feeders and bulk feeders employ some degree of suction during the feeding cycle [1,2,32], suggesting that the use of suction is probably ancestral in crown Cetacea. It is, therefore, critical to distinguish between raptorial feeders capable of producing suction and true suction specialists.

Interpreting suction feeding as the ancestral condition for aetiocetids based on NMV P252567 is problematic as the dental wear indicative of suction feeding is not observed in any other known aetiocetid. Instead, we consider it likely that some degree of suction is ancestral both for crown Cetacea and for stem Mysticeti. However, we consider the morphology of NMV P252567 a derived condition reflecting a suction specialist.

#### 5.3.3. Proto-baleen

It has been suggested that palatal foramina medial to the tooth alveoli of some aetiocetids are homologous to baleen innervation and vascularization in extant mysticetes, suggesting the presence of proto-baleen [8,12]. Recent authors have questioned the viability of this hypothesis [11,13,14]. This hypothesis requires that proto-baleen is the ancestral condition for aetiocetids, otherwise it represents a homoplastic condition suggesting that baleen evolved twice: within aetiocetids and again in crown Mysticeti [11]. However, palatal foramina are present in only three species of aetiocetids (*A. cotylalveus*, *A. weltoni* and *F. goedertorum*). Moreover, aetiocetids such as *F. buelli* and *S. meadi* preserve evidence of tight dental occlusion that would have been hindered by proto-baleen [11]. Therefore, the presence of teeth and proto-baleen simultaneously has no known analogy from a biomechanical standpoint; this hypothesis is neither the most parsimonious phylogenetic explanation, nor has it been demonstrated to be biomechanically viable.

#### 5.3.4. The challenges of reconstructing ancestral feeding morphologies

The disparity in feeding morphologies across aetiocetids creates challenges with reconstructing the ancestral condition of any trait leading to crown Mysticeti, in light of this phylogenetic analysis and subsequent taxonomic revision. The ‘weakly heterodont' dentition of *Aetiocetus* is restricted to that genus, suggesting that this simplification is a derived trait unrelated to the evolution of tooth loss. NMV P252567 [13] clearly exhibits morphology specialized for suction, but the absence of this morphology in other aetiocetids suggests that it is a derived trait. *Salishicetus meadi* and *F. buelli* each represent aetiocetids with precise occlusion incompatible with the spacing needed for putative proto-baleen. This finding, in addition to the questionable homology of palatal foramina relative to palatal sulci housing baleen in living mysticetes [14] and the unknown feeding biomechanics of a gape cycle using proto-baleen and teeth [11,14], make the hypothesis of proto-baleen in aetiocetids dubious.

## 6. Conclusion

In the light of this evidence, we consider the most likely ancestral feeding state for Aetiocetidae to be suction-assisted macrophagous raptorial feeding. This foraging behaviour is similar to that of their stem cetacean ancestors [33], other stem mysticetes [31], and probably reflects the condition ancestral of crown Cetacea [1,2]. Accordingly, we interpret the simplified dentition of *Aetiocetus* spp. and the suction specializations of NMV P252567 as derived conditions specific to their respective lineages and see no basis for inferring the presence of proto-baleen. Any alternate interpretation suggests one or more homoplastic characters and is, therefore, not parsimonious. Given this interpretation, we suggest that aetiocetid feeding morphologies are unlikely to shed new light on the evolution of baleen, and suggest that such answers lie closer to crown Mysticeti.

## Supplementary Material

Figure S1

## Supplementary Material

Figure S2

## Supplementary Material

Figure S3

## Supplementary Material

Figure S4

## Supplementary Material

Salishicetus Final Matrix

## Data accessibility

The following information was supplied regarding data availability: Zenodo: (doi:10.5281/zenodo.834321)
