# Supplementary figures and images for: Salishicetus meadi, a new aetiocetid from the late Oligocene of Washington State and implications for feeding transitions in early mysticete evolution
Source: R Soc Open Sci. 2018 Apr 18;5(4):172336. doi: 10.1098/rsos.172336 (PMC5936946; doi:10.1098/rsos.172336)

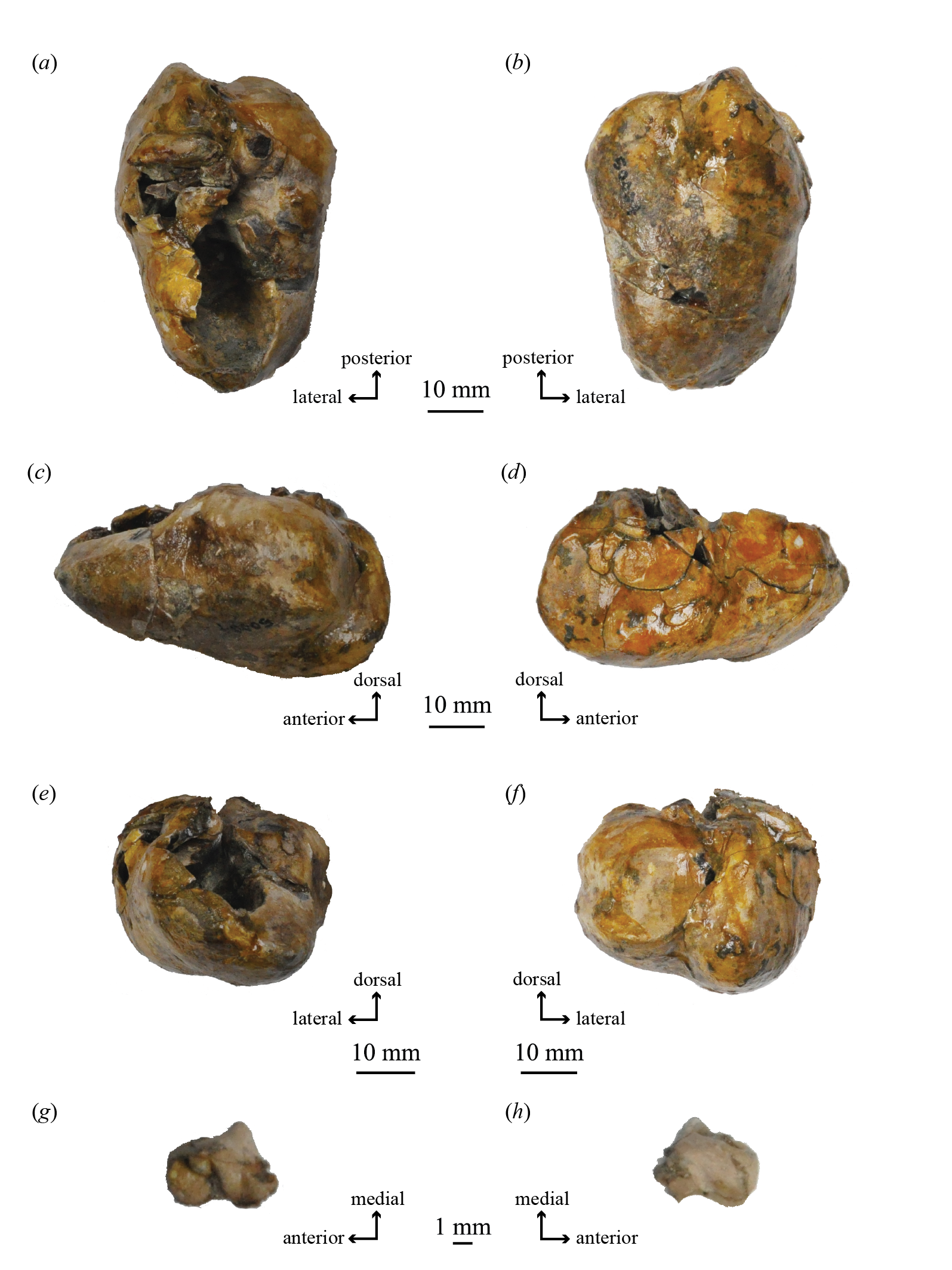

Supplement: Figure S1 [file rsos172336supp1.tif]

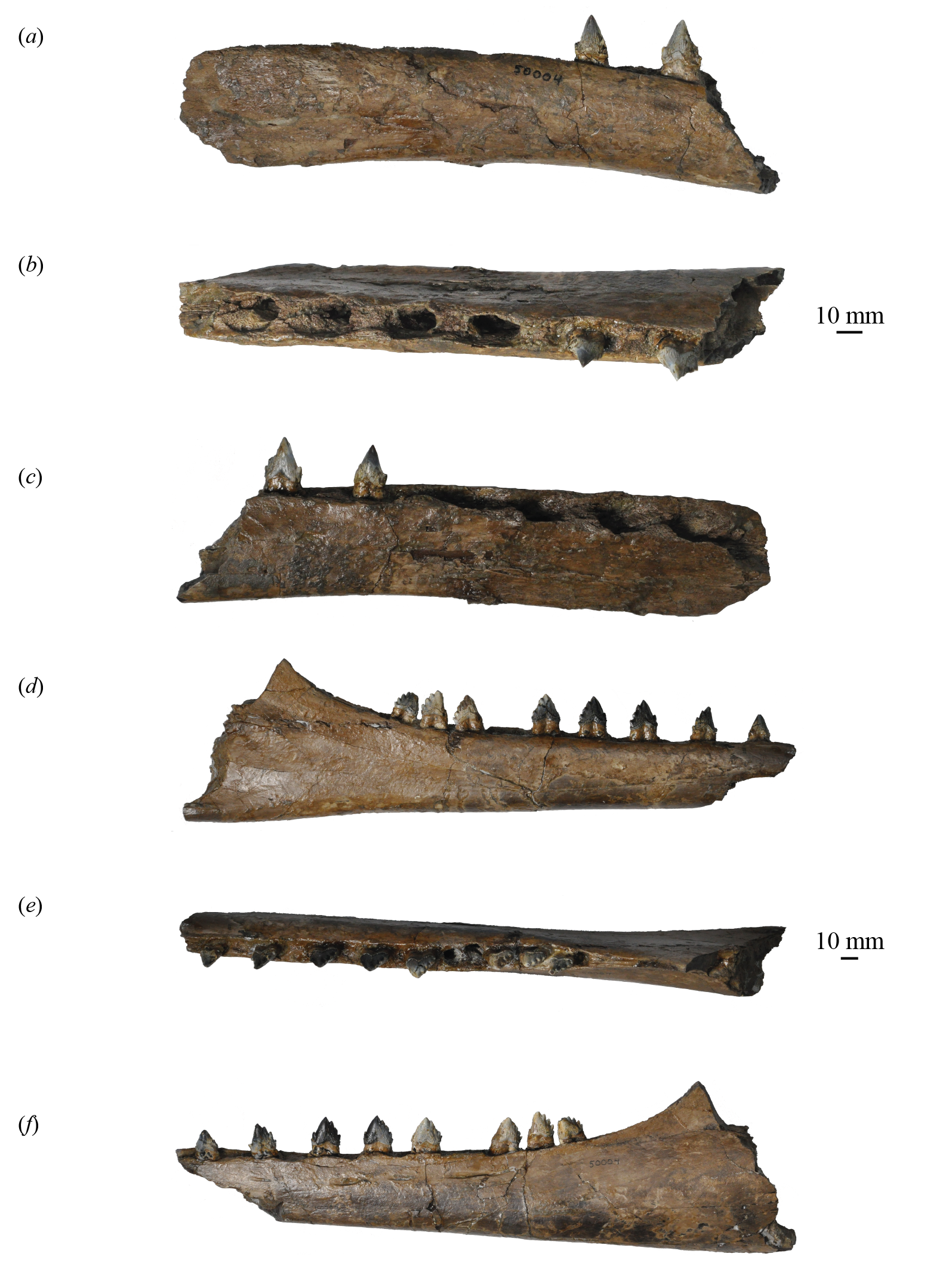

Supplement: Figure S2 [file rsos172336supp2.tif]

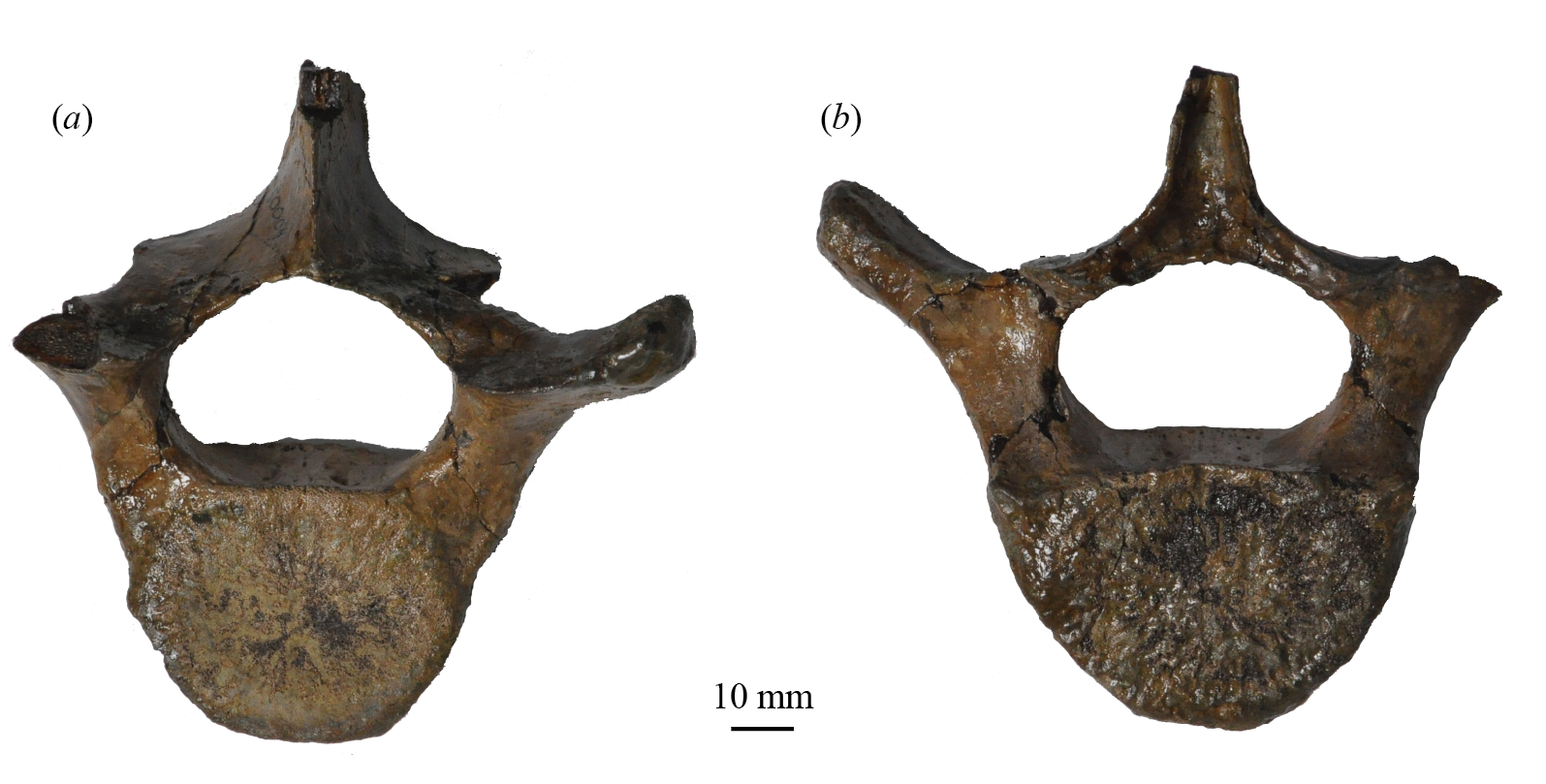

Supplement: Figure S3 [file rsos172336supp3.tif]

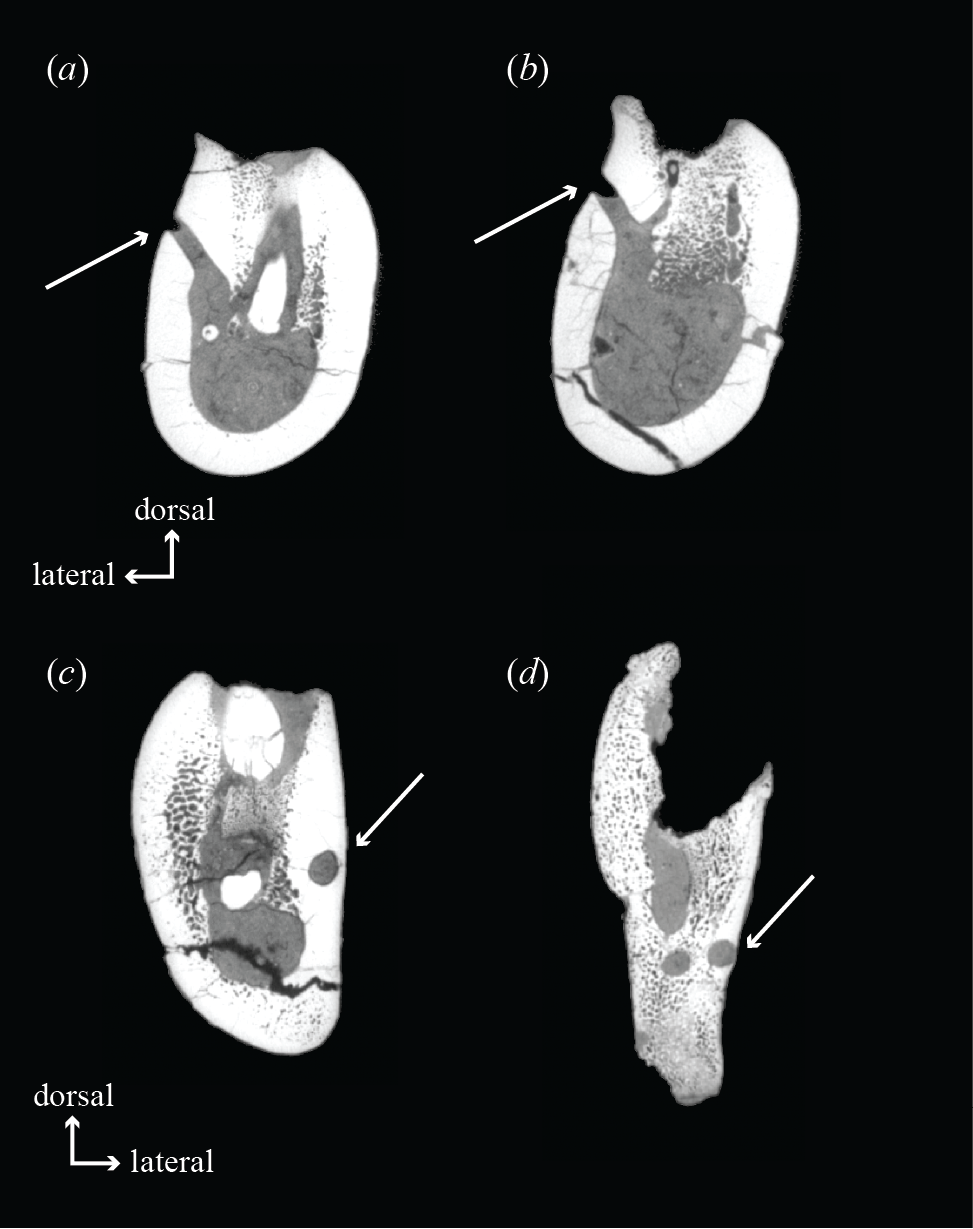

Supplement: Figure S4 [file rsos172336supp4.tif]
